# Supplementary material for: Galectin-9 restricts hepatitis B virus replication via p62/SQSTM1-mediated selective autophagy of viral core proteins
Source: Nat Commun. 2022 Jan 27;13:531. doi: 10.1038/s41467-022-28171-5 (PMC8795376; doi:10.1038/s41467-022-28171-5)
Supplement: Supplementary file 1 — Supplementary Information [file 41467_2022_28171_MOESM1_ESM.pdf]

Supplementary Information

Supplementary Figure 1

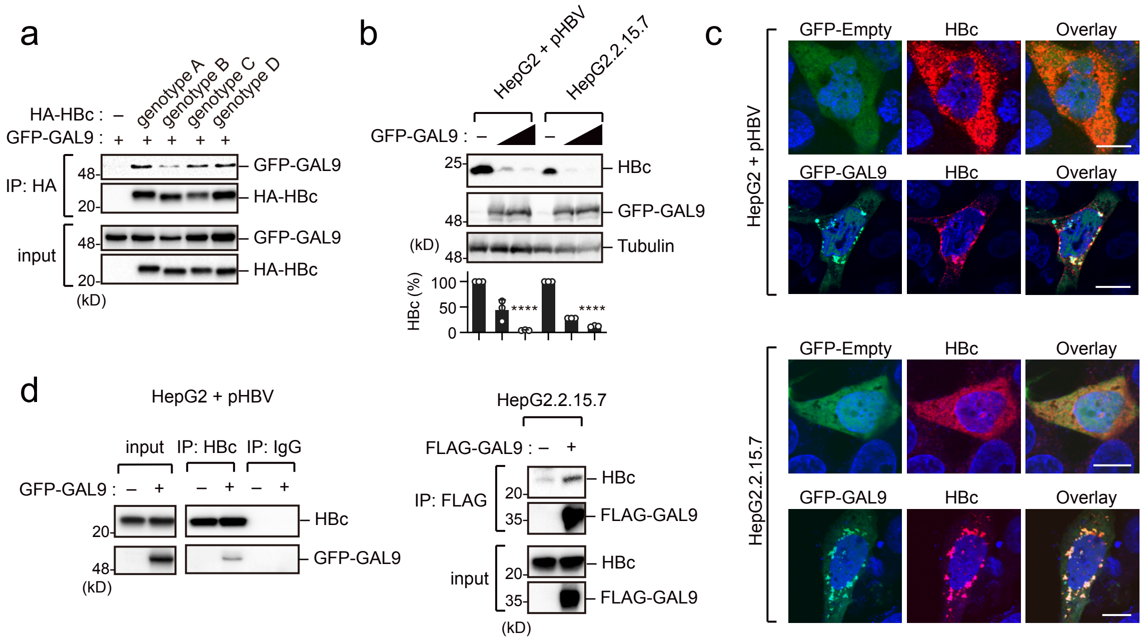

Supplementary Figure 1. Effects of GAL9 in HepG2 cells expressing HBV genome
(a) GAL9 can interact with HBc in hepatocytes. Immunoprecipitation assays of HepG2 cells expressing HA-HBc (derived from HBV genotypes A–D) and GFP-GAL9. Cell lysates were precipitated with anti-HA antibody, followed by immunoblotting.
(b) GAL9 reduces HBc expression. HBV DNA (genotype C)-transduced HepG2 cells or HepG2.2.15.7 cells were transfected with vector encoding GFP-GAL9. Cells were subjected to immunoblotting analysis to detect the indicated proteins. Bar chart indicates the ratio of HBc over Tubulin, as determined by densitometry, and is presented as a mean ± SD (n = 3 experiments). ****$P < 0.0001$, two-tailed unpaired t-test.
(c) GAL9 and HBc accumulation in cytoplasmic bodies. Confocal microscopic imaging of HBV DNA (genotype C)-transduced HepG2 cells or HepG2.2.15.7 cells expressing GFP-GAL9. Nuclei were stained with DAPI. Scale bar, 10 μm.
(d) Immunoprecipitation assays of HBV DNA (genotype C)-transduced HepG2 cells or HepG2.2.15.7 cells expressing GFP-GAL9 or FLAG-GAL9. Cell lysates were precipitated with anti-HBc or anti-FLAG antibody, followed by immunoblotting.
Immunoblots and micrographs are representative of experiments with similar results ($n \geq 2$). Source data are provided as a Source Data file.

Supplementary Figure 2

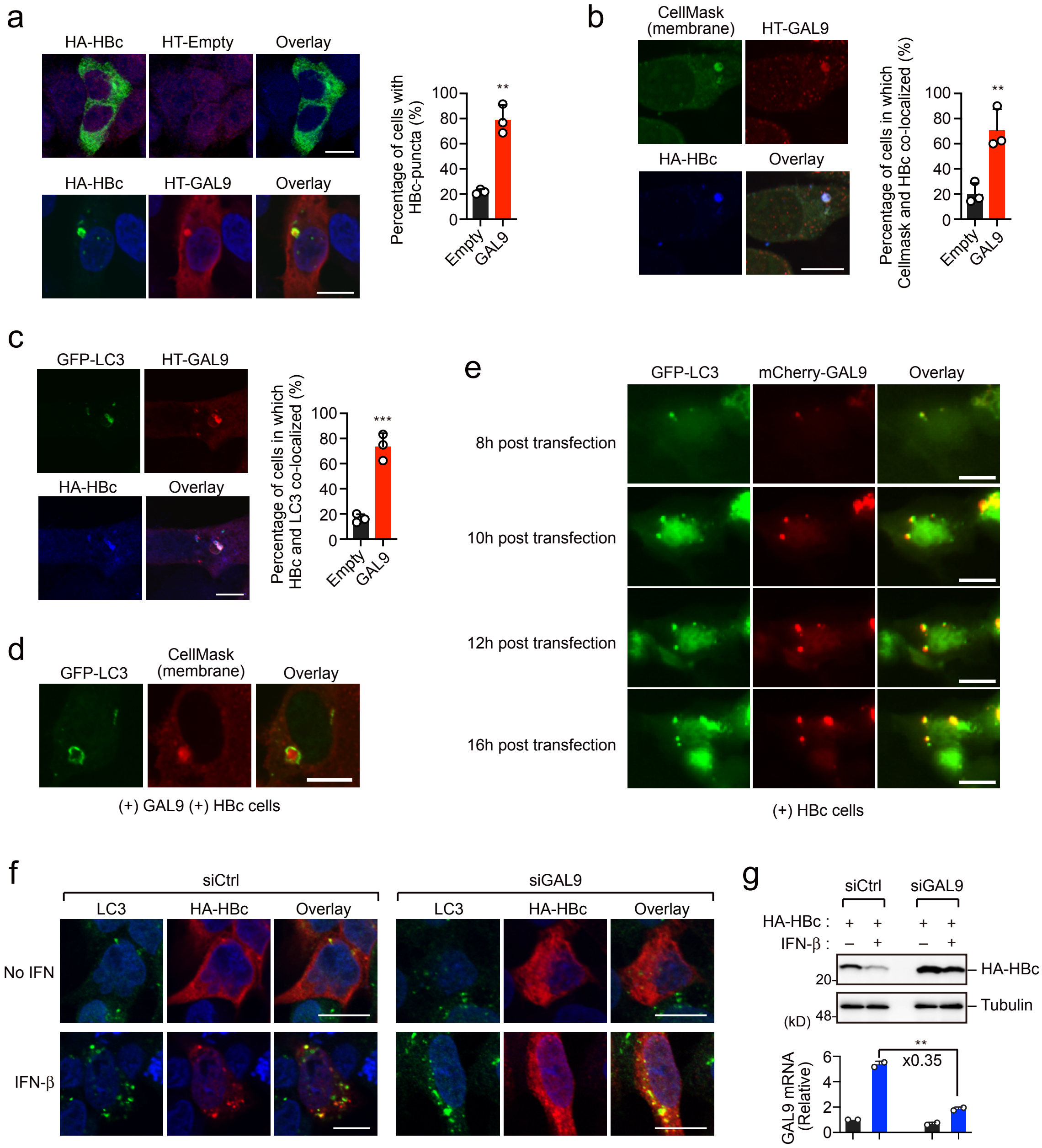

Supplementary Figure 2. GAL9 leads to HBc aggregation

(a) Another view of the cell image in Figure 2d. The graph on the right is the percentage of cells with aggregated HBc (n = 50 cells examined over three experiments, mean ± SD). **P = 0.0011, two-tailed unpaired t-test. Scale bar, 10 µm.

(b) Another view of the cell image in Figure 2e. The graph on the right is the percentage of cells in which Cellmask staining and HBc co-localized (n = 50 cells examined over three experiments, mean ± SD). **P = 0.0094, two-tailed unpaired t-test. Scale bar, 10 µm.

(c) Another view of the cell image in Figure 2f. The graph on the right is the percentage of cells in which LC3 and HBc co-localized (n = 50 cells examined over three experiments, mean ± SD). ***P = 0.0008, two-tailed unpaired t-test. Scale bar, 10 µm.

(d) Representative image of Cellmask and LC3 staining of HepG2 cells co-expressing GAL9 and HBc. Scale bar, 10 μm.
(e) Live cell imaging of HepG2 cells expressing GFP-LC3 (green), mCherry-GAL9 (red), and HBc. Scale bar, 10 μm.
(f) Another view of the cell image in Figure 2g. The graph on the right is the percentage of cells in which HBc localized to LC3-positive puncta. Scale bar, 10 μm.
(g) HBc levels are decreased by IFN-β, but are restored by GAL9 depletion. Immunoblotting analysis was performed on HepG2 cells expressing HA-HBc in the presence or absence of IFN-β (1000 U/mL). GAL9 mRNA levels are also shown. The mean ± SD of two independent determinations is plotted. $^{**}P = 0.0039$, two-tailed unpaired t-test.
Immunoblots and micrographs are representative of experiments with similar results ($n \geq 2$). Source data are provided as a Source Data file.

Supplementary Figure 3

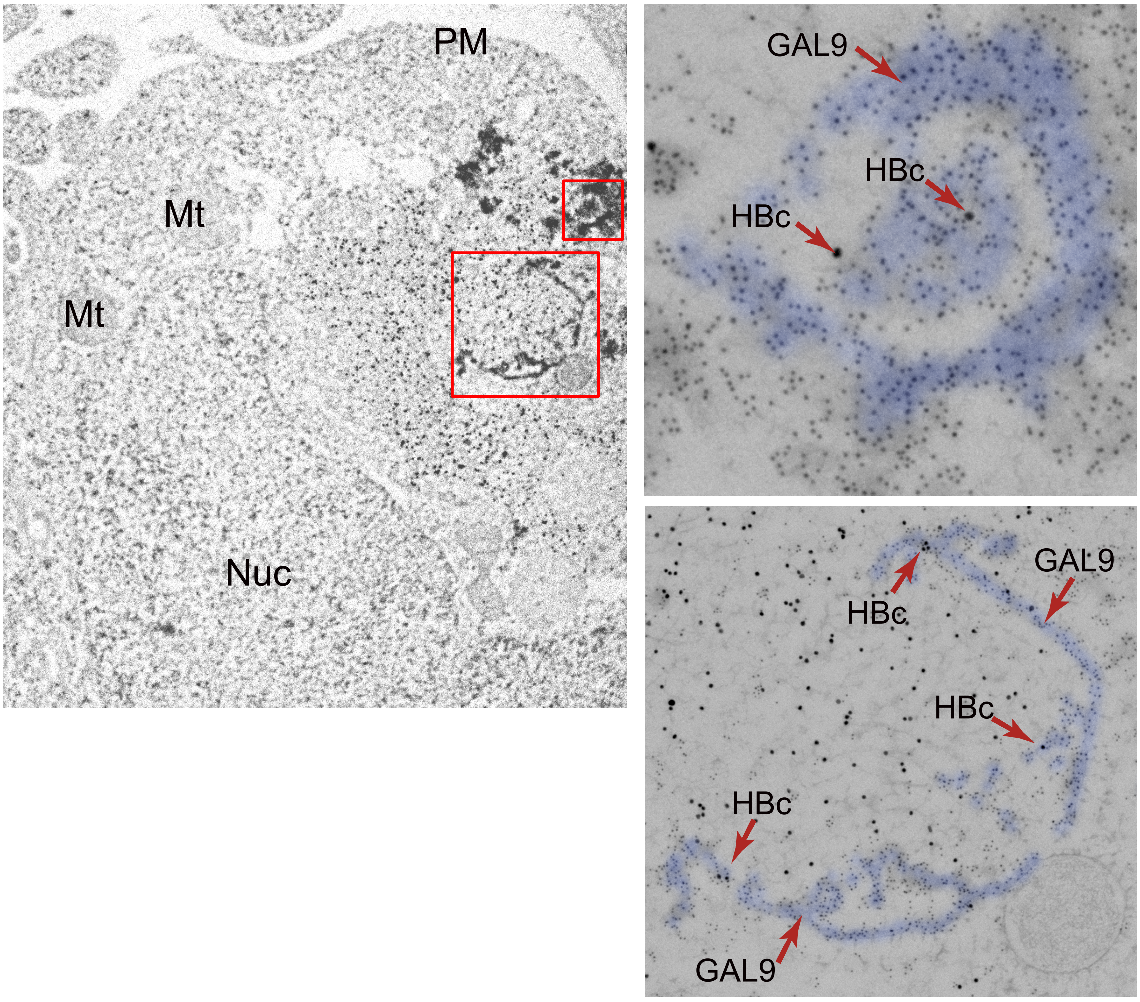

Supplementary Figure 3. Immune-electromicroscopy of GAL9-mediated HBc aggregation

Immune-electromicroscopic analysis of HepG2 cells expressing GFP-GAL9 and HA-HBc. Cells were fixed and stained with anti-GFP (10 nm particles) and anti-HA (20 nm particles) antibodies. Expanded views are also shown. Areas showing membrane structures are colored blue. Micrographs are representative of experiments with similar results ($n = 2$). Nuc, nucleus; Mt, mitochondria; PM, plasma membrane

Supplementary Figure 4

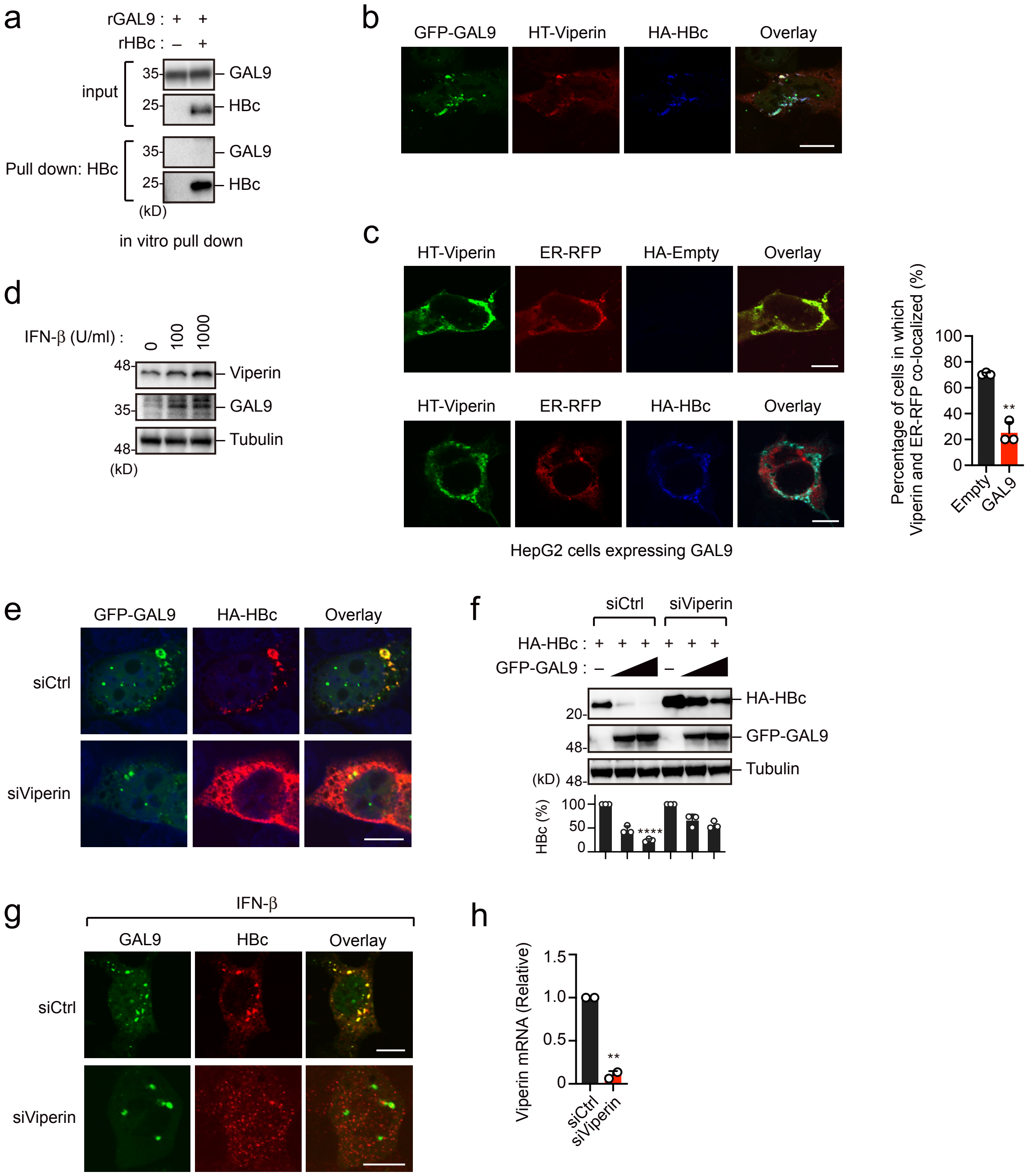

Supplementary Figure 4. GAL9 does not directly bind HBc

(a) GAL9 does not bind directly to HBc. Recombinant HBc and GAL9 were incubated and subjected to in vitro pull-down assay using anti-HBc antibody.

(b) Another view of the cell image in Figure 3c.

(c) ER-localized viperin is depleted in cells expressing HBc. Confocal microscopic imaging of HepG2 cells expressing GAL9, HT-viperin, and HA-HBc. Cells were treated with ER-RFP reagent 24 h prior to staining. The graph on the right is the percentage of cells in which viperin and ER-RFP co-localized (n = 50 cells examined over three experiments, mean ± SD). **P = 0.0008, two-tailed unpaired t-test. Scale bar, 10 μm.

(d) IFN triggers viperin and GAL9 expression. HepG2 cells were treated with IFN-β (0, 100, 1000 U/mL) for 24 h and subjected to immunoblotting.
(e) Another view of the cell image in Figure 3g.
(f) Viperin is a cofactor for GAL9 activity. Immunoblotting analysis of HepG2 cells expressing HA-HBc and GFP-GAL9. Cells were transduced with siRNA targeting viperin at 24 h prior to DNA transfection. Bar chart indicates the ratio of HBc over Tubulin, as determined by densitometry, and is presented as a mean ± SD (n = 3 experiments). ****$P < 0.0001$, two-tailed unpaired t-test.
(g) Another view of the cell image in Figure 3h.
(h) Viperin knockdown efficiency confirmed by RT-PCR. The mean ± SD of two independent determinations is plotted. **$P = 0.0016$, two-tailed unpaired t-test.

Immunoblots and micrographs are representative of experiments with similar results ($n \geq 2$). Source data are provided as a Source Data file.

Supplementary Figure 5

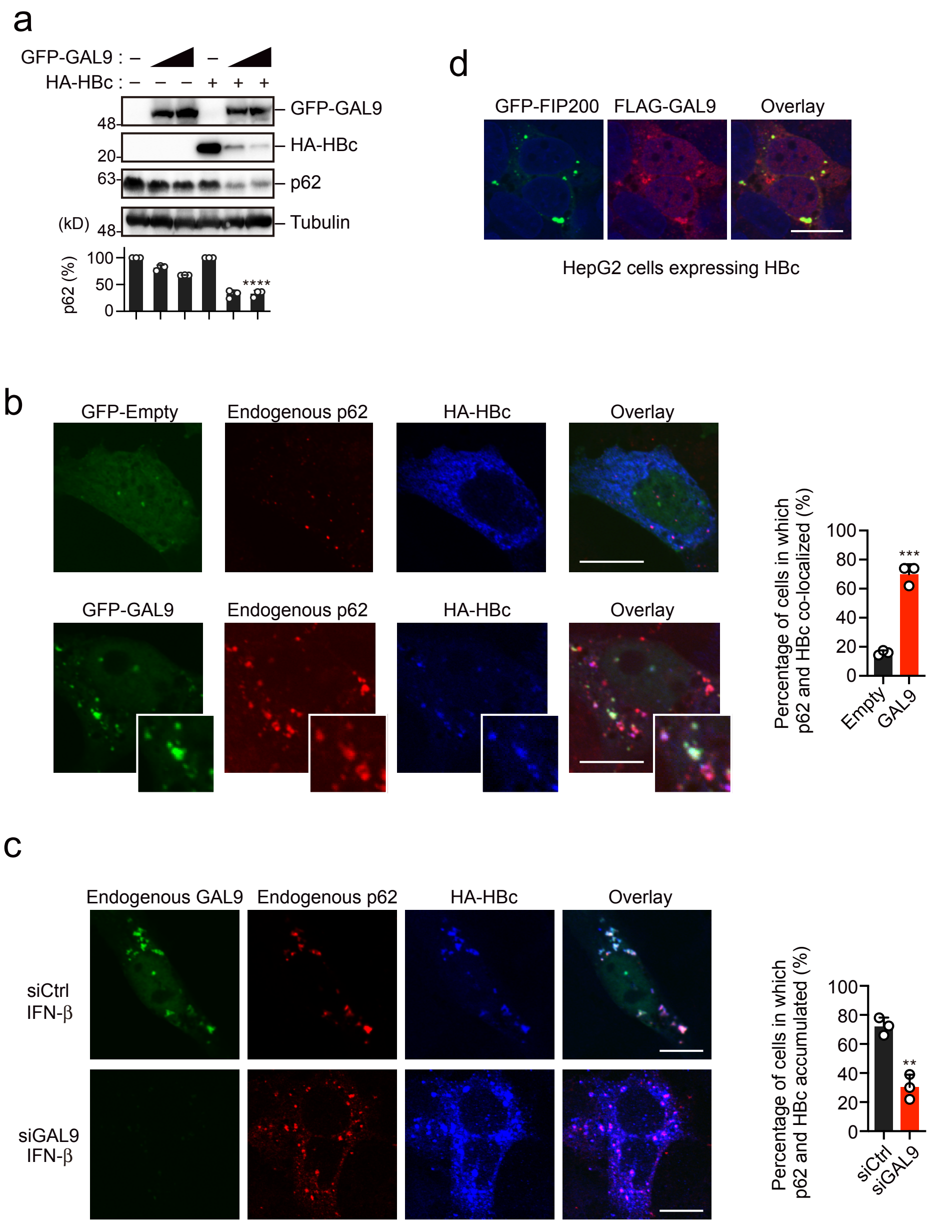

Supplementary Figure 5. GAL9 acts on selective autophagy targeting HBc
(a) GAL9 acts on selective autophagy targeting HBc and, to a lesser extent, on pan-autophagy. HepG2 cells were transfected with vector encoding GFP-GAL9 with or without HA-HBc. Cells were subjected to immunoblotting analysis to detect the indicated proteins. Bar chart indicates the ratio of p62 over Tubulin, as determined by densitometry, and is presented as a mean ± SD (n = 3 experiments). ****$P < 0.0001$, two-tailed unpaired t-test.
(b) Another view of the cell image in Figure 4c. The graph on the right is the percentage of cells in which p62 and HBc co-localized (n = 50 cells examined over three experiments, mean ± SD). ***$P = 0.0002$, two-tailed unpaired t-test. Scale bar, 10 µm.

(c) GAL9 involves the IFN-induced accumulation of HBc and p62. HepG2 cells, transduced with control- (Ctrl) or GAL9-targeting siRNA, were transfected with vectors encoding HA-HBc. Cells were then treated with IFN-β (1000 U/mL) for 24 h. The graph on the right shows the percentage of cells in which p62 and HBc are co-localized (n = 50 cells examined over three experiments, mean ± SD). **P = 0.0023, two-tailed unpaired t-test. Scale bar, 10 μm.
(d) GAL9 colocalizes with FIP200 in HBc-expressing HepG2 cells. Confocal microscopic imaging of HepG2 cells expressing GFP-FIP200 (green), FLAG-GAL9 (red), and HBc. Nuclei were stained with DAPI. Scale bar, 10 μm.
Immunoblots and micrographs are representative of experiments with similar results ($n \geq 2$). Source data are provided as a Source Data file.

Supplementary Figure 6

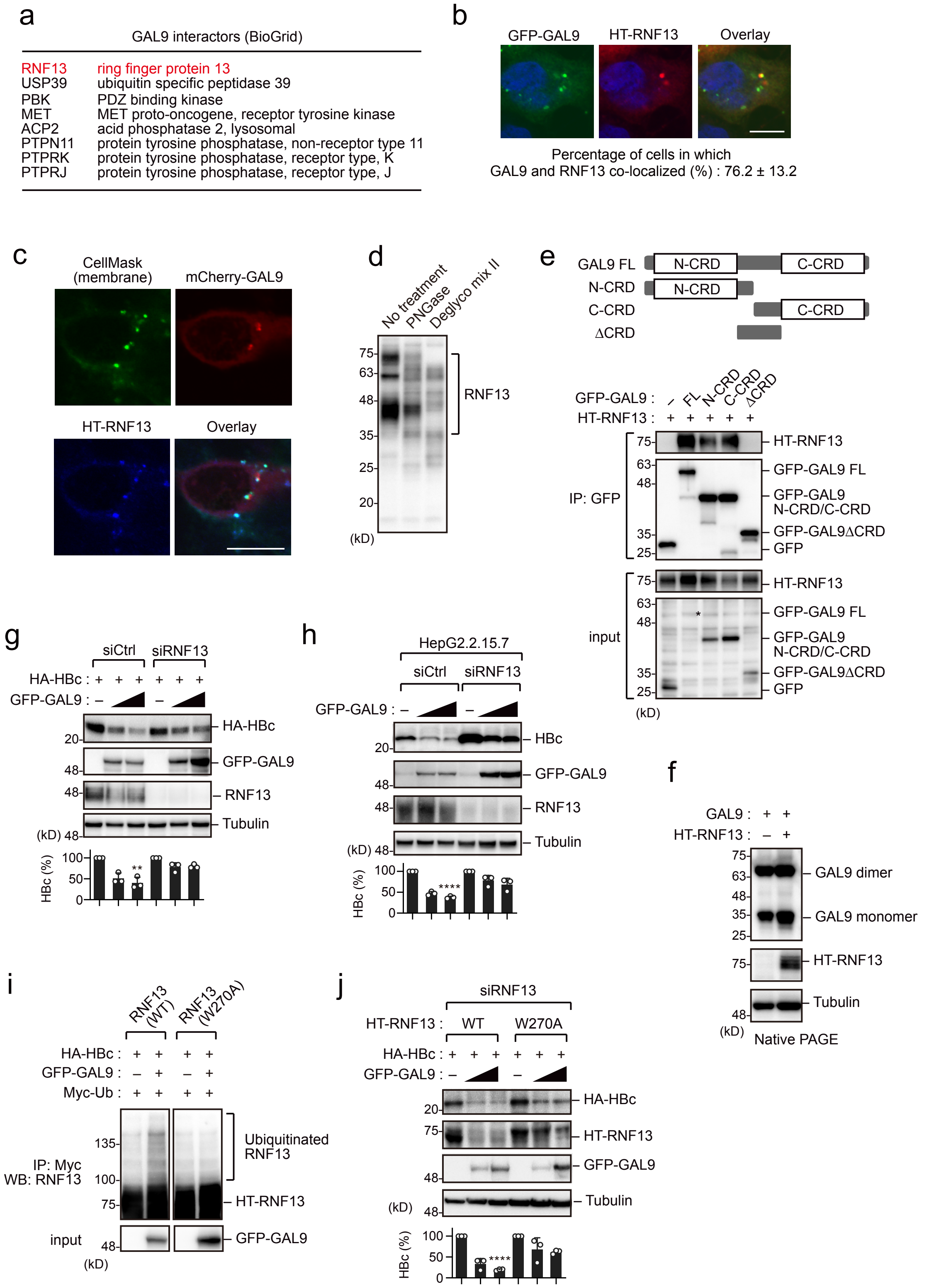

Supplementary Figure 6. GAL9 leads to RNF13 ubiquitination

(a) Assembly of known GAL9 interactors that are involved in post-translational modifications from the BioGrid database.

(b) Another view of the cell image in Figure 5C. The graph on the right is the percentage of cells in which GAL9 co-localized with RNF13. At least 50 randomly selected cells were analyzed. Scale bar, 10 μm.
(c) Colocalization of GAL9 and RNF13 on membrane structures. Representative images of HepG2 cells stained with CellMask, mCherry-GAL9, and HT-RNF13. Scale bar, 10 μm.
(d) RNF13 is glycosylated in hepatocytes. HepG2 cells expressing HT-RNF13 were lysed and incubated with PNGase F (5 U/μL) or Protein Deglycosylation Mix II (5 units/μL) at 37°C for 1 h.
(e) RNF13 binds GAL9 CRDs. Immunoprecipitation assays of HepG2 cells expressing GFP-GAL9 mutants (N-CRD, C-CRD, and ΔCRD) and HT-RNF13. Cell lysates were precipitated with anti-GFP antibodies, followed by immunoblotting.
(f) RND13 does not inhibit dimerization of GAL9. Native PAGE analysis of cell lysates expressing GAL9 with or without HT-RNF13.
(g, h) RNF13 is a cofactor for GAL9 activity. Immunoblotting analysis of HepG2 cells expressing HA-HBc and GFP-GAL9 **(g)** or HepG2.2.15.7 cells expressing GFP-GAL9 **(h)**. Cells were transduced with siRNA targeting RNF13 at 24 h prior to DNA transfection.
(i) GAL9 promotes auto-ubiquitination of RNF13. HepG2 cells expressing HA-HBc, GFP-GAL9, Myc-Ub, and RNF13 (WT or W270A) were lysed and precipitated with anti-Myc antibody, followed by immunoblotting with anti-RNF13.
(j) Auto-ubiquitination activity of RNF13 is required for GAL9 activity. HepG2 cells were transduced with RNF13-targeting siRNA, and then transfected with vectors expressing HA-HBc, GFP-GAL9, and siRNA-resistant HT-RNF13 (WT or W270A). Cells were subjected to immunoblotting analysis to detect the indicated proteins.
Bar charts in **(g, h, j)** indicate the ratio of HBc over Tubulin or Vinculin, as determined by densitometry, and are presented as a mean ± SD (n = 3 experiments). $^{**}P = 0.0018$ **(g)**, $^{****}P < 0.0001$ **(h, j)**, two-tailed unpaired t-test.
Immunoblots and micrographs are representative of experiments with similar results ($n \geq 2$). Source data are provided as a Source Data file.

Supplementary Figure 7

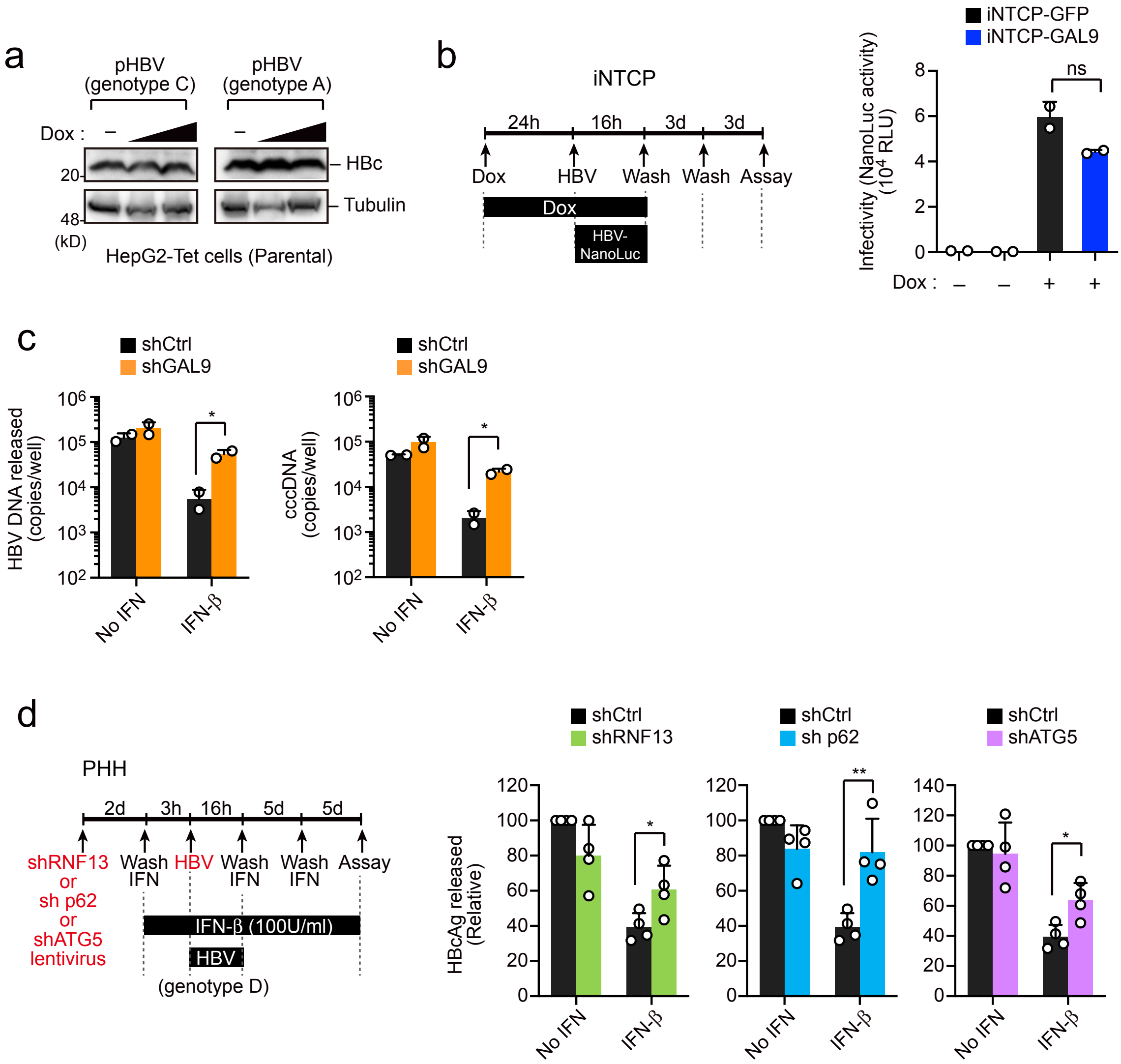

Supplementary Figure 7. GAL9 and its cofactors play a role in the anti-HBV activity of IFN

(a) HepG2-Tet cells (GAL9 non-inducible) were transfected with pHBV genotype A or C, and then were treated with doxycycline (0.5 or 1 µg/mL). Cells were subjected to immunoblotting analysis to detect the indicated proteins.

(b) GAL9 only modestly inhibits viral entry. The experimental design is shown on the left. HepG2-iNTCP cells stably expressing GFP or GFP-GAL9 were infected with HBV-NanoLuc reporter virus in the presence of Dox. Dox induces expression of the HBV entry receptor NTCP. Seven days after infection, cell lysates were subjected to NanoLuc assay to measure the efficiency of the early phase of HBV infection. The mean ± SD of two independent determinations is plotted. P = 0.6728 (ns; not significant), two-tailed unpaired t-test.

(c) GAL9 depletion attenuates antiviral activity of IFN. The experimental design is shown in Figure 6c. Eleven days after infection, supernatants were subjected to qPCR (left) to detect released infectious HBV. Cells were subjected to qPCR to detect

cccDNA (right). Bar charts are presented as a mean ± SD (n = 3 experiments). *P = 0.0179 (left), *P = 0.0393 (right), two-tailed unpaired t-test.

(d) RNF13, p62, and ATG5 depletion attenuates antiviral activity of IFN. The experimental design is shown on the left. Eleven days after infection, cell supernatants were subjected to HBcAg ELISA to detect released HBV. Bar charts are presented as a mean ± SD (n = 4 experiments). *P = 0.0391 (left), **P = 0.0063 (middle), *P = 0.0145 (right), two-tailed unpaired t-test.

Immunoblots and micrographs are representative of experiments with similar results ($n \geq 2$). Source data are provided as a Source Data file.

Supplementary Table 1. Sequences of primers used in this study.

Target	Primer pair (5' - 3')
**HBV DNA (HBc)**	GAGTGTGGATTCGCACTCC
	GAGGCGAGGGAGTTCTTCT
**cccDNA**	CGTCTGTGCCTTCTCATCTGC
	GCACAGCTTGGAGGCTTGAA
**ACTB**	GGACTTCGAGCAAGAGATGG
	AGCACTGTGTTGGCGTACAG
**LGALS9**	ACACCCAGATCGACAACTCCTG
	CAAACAGGTGCTGACCATCCAC

Supplementary Table 2. Antibodies used in this study.

Antibody	Source	Dilution
**HA**	MBL #M180-3S	1:1000 (WB)<br>1:100 (IF)
**GFP**	MBL #598	1:1000
**FLAG**	Merck #F3165 (WB)<br>Merck #F7425 (IF)	1:5000 (WB)<br>1:100 (IF)
**HaloTag**	Promega #G9211 (WB)<br>Promega #G9281 (IF)	1:1000 (WB)<br>1:100 (IF)
**Myc**	CST #2276S	1:1000 (WB)<br>1:100 (IF)
**α-Tubulin**	Merck #T6199	1:5000
**vinculin**	Merck #V9131	1:5000
**GAL9**	R&D Systems #AF2045	1:1000
**p62/SQSTM1**	MBL #PM045Y	1:1000
**LC3**	MBL #PM036Y	1:1000
**RNF13**	Merck #HPA064784	1:1000
**Ubiquitin**	Santa Cruz #sc-8017	1:1000
**UBE1**	Santa Cruz #sc-53555	1:1000
**HBc**	Kanto Chemical (clone 7B2)	1:1000
**HBs**	In-house (PMID: 32105634)	1:1000
**Alexa488-conjugated anti-mouse IgG**	Thermo #A11029	1:500
**Alexa568-conjugated anti-rabbit IgG**	Thermo #A10042	1:500
**Alexa405-conjugated anti-mouse IgG**	Thermo #A31553	1:500
**CellMask Green membrane stain**	Thermo #C37608	1:500
**CellLight ER-RFP, BacMam 2.0**	Thermo #C10591	2 µL per 10,000 cells
**HRP-conjugated anti-mouse IgG**	Cytiva #NA931	1:10000
**HRP-conjugated anti-rabbit IgG**	Cytiva #NA934	1:10000
**HRP-conjugated anti-goat IgG**	Merck #AP106P	1:10000

Supplementary Table 3. Recombinant proteins used in this study.

Recombinant proteins	Source
**GAL9**	Wako 074-06421
**HBc**	Merck #H8909
**Viperin**	Novus Biologicals #H00091543
**UBE1**	Merck #SRP6147
**UBcH5c**	Merck #662098
**Ubiquitin**	Merck #U5507

Supplementary methods

Immunoelectron microscopy

Cells expressing HA-HBc and GFP-GAL9 on the gold disks were frozen in liquid propane at -175°C and then freeze substituted with 2% tannic acid in ethanol and 2% distilled water at -80°C for 48 h. Cells were then transferred at -20°C for 3 h and warmed up to 4°C for 1 h. The samples were dehydrated with anhydrous ethanol, infiltrated with an ethanol/resin mixture (LR white; London Renin) at 4°C for 30 min, transferred to a fresh 100% resin, and polymerized at 50°C overnight. The polymerized resins were ultra-thin sectioned at 70 nm by using an ultramicrotome (Ultracut UCT), and the sections were mounted on nickel grids. The grids were incubated with anti-HA (MBL) and anti-GFP (Abcam) antibodies at 4°C overnight. The grids were washed with PBS containing 1% BSA and incubated with anti-mouse IgG conjugated to 20 nm gold particles (BBI Solutions) and anti-rabbit IgG conjugated to 10 nm gold particles (BBI Solutions) at 27°C for 2 h. The grids were dried and stained with 2% uranyl acetate for 10 min and in Lead stain solution (Sigma) for 3 min. Imaging was performed using a transmission electron microscope (JEM-1400Plus, JEOL) at an acceleration voltage of 100 kV.
