# Supplementary material for: Galectin-9 restricts hepatitis B virus replication via p62/SQSTM1-mediated selective autophagy of viral core proteins
Source: Nat Commun. 2022 Jan 27;13:531. doi: 10.1038/s41467-022-28171-5 (PMC8795376; doi:10.1038/s41467-022-28171-5)
Supplement: Supplementary file 3 — Reporting Summary [file 41467_2022_28171_MOESM3_ESM.pdf]

Corresponding author(s): Akihide Ryo

Last updated by author(s): December 14, 2021

Reporting Summary

Nature Research wishes to improve the reproducibility of the work that we publish. This form provides structure for consistency and transparency in reporting. For further information on Nature Research policies, see our Editorial Policies and the Editorial Policy Checklist.

Statistics

For all statistical analyses, confirm that the following items are present in the figure legend, table legend, main text, or Methods section.

n/a	Confirmed	
☐	☒	The exact sample size (*n*) for each experimental group/condition, given as a discrete number and unit of measurement
☒	☐	A statement on whether measurements were taken from distinct samples or whether the same sample was measured repeatedly
☐	☒	The statistical test(s) used AND whether they are one- or two-sided<br>*Only common tests should be described solely by name; describe more complex techniques in the Methods section.*
☒	☐	A description of all covariates tested
☒	☐	A description of any assumptions or corrections, such as tests of normality and adjustment for multiple comparisons
☐	☒	A full description of the statistical parameters including central tendency (e.g. means) or other basic estimates (e.g. regression coefficient) AND variation (e.g. standard deviation) or associated estimates of uncertainty (e.g. confidence intervals)
☐	☒	For null hypothesis testing, the test statistic (e.g. *F*, *t*, *r*) with confidence intervals, effect sizes, degrees of freedom and *P* value noted<br>*Give P values as exact values whenever suitable.*
☒	☐	For Bayesian analysis, information on the choice of priors and Markov chain Monte Carlo settings
☒	☐	For hierarchical and complex designs, identification of the appropriate level for tests and full reporting of outcomes
☒	☐	Estimates of effect sizes (e.g. Cohen's *d*, Pearson's *r*), indicating how they were calculated

Our web collection on statistics for biologists contains articles on many of the points above.

Software and code

Policy information about availability of computer code

Data collection	GloMax® Discover System Software 3.2.3, ATTO ImageSaver 6.0
Data analysis	ImageJ 1.4, Prism 8

For manuscripts utilizing custom algorithms or software that are central to the research but not yet described in published literature, software must be made available to editors and reviewers. We strongly encourage code deposition in a community repository (e.g. GitHub). See the Nature Research guidelines for submitting code & software for further information.

Data

Policy information about availability of data

All manuscripts must include a data availability statement. This statement should provide the following information, where applicable:

- Accession codes, unique identifiers, or web links for publicly available datasets
- A list of figures that have associated raw data
- A description of any restrictions on data availability

The source data underlying Figs.1a-d, 2a-c, g, 3a, b, d-h, 4a, b, d, e, 5a, b, d-h, 6a, b, d-f, h, i, and Supplementary Figs. 1a, b, d, 2a-c, 2g, 4a, c-e, h, 5a-c, 6d-j, and 7a-d are provided as a Source Data file.

Field-specific reporting

Please select the one below that is the best fit for your research. If you are not sure, read the appropriate sections before making your selection.

☒ Life sciences ☐ Behavioural & social sciences ☐ Ecological, evolutionary & environmental sciences

For a reference copy of the document with all sections, see nature.com/documents/nr-reporting-summary-flat.pdf

Life sciences study design

All studies must disclose on these points even when the disclosure is negative.

Sample size	Experiments have repeated a multiple of at least three times. Three independent biological experiments are sufficient to ensure data reproducibility and P value calculation. P values were calculated with a two-sided unpaired t-test if without specification.
Data exclusions	No data were excluded from analysis.
Replication	The initial screening using NanoBRET shown in Fig.1A has been performed in triplicate for each sample. Western blot analysis, microscopic analysis, and HBV infection assays were repeated at least three times independently. We specified the number of replications in the Figure legends. All replication attempts were successful.
Randomization	There was no randomization for experiments as this study does not include experiments with animals and clinical samples.
Blinding	Investigators assessing quantification using cell images were blinded. For all other experiments, blinding was not necessary because they did not involve subjective evaluation.

Reporting for specific materials, systems and methods

We require information from authors about some types of materials, experimental systems and methods used in many studies. Here, indicate whether each material, system or method listed is relevant to your study. If you are not sure if a list item applies to your research, read the appropriate section before selecting a response.

Materials & experimental systems

n/a	Involved in the study	
☐	☒	Antibodies
☐	☒	Eukaryotic cell lines
☒	☐	Palaeontology and archaeology
☒	☐	Animals and other organisms
☒	☐	Human research participants
☒	☐	Clinical data
☒	☐	Dual use research of concern

Methods

n/a	Involved in the study	
☒	☐	ChIP-seq
☒	☐	Flow cytometry
☒	☐	MRI-based neuroimaging

Antibodies

Antibodies used	All antibodies and dilutions used in this study were shown in Supplementary Table 2.
Validation	The following antibodies in this study were commercially purchased and have been validated by the vendors. Validation data are available from the vendor's respective websites.<br>anti-HA (MBL, #M180-3S) https://www.mblintl.com/products/m180-3/<br>anti-GFP (MBL, #598) https://www.mblintl.com/products/598/<br>anti-FLAG (Merck, #F3165) https://www.sigmaaldrich.com/US/en/product/sigma/f3165<br>anti-FLAG (Merck, #F7425) https://www.sigmaaldrich.com/US/en/product/sigma/f7425<br>anti-HaloTag (Promega, #G9211) https://ch.promega.com/products/protein-detection/primary-and-secondary-antibodies/anti-halotag-monoclonal-antibody/<br>anti-HaloTag (Promega, #G9281) https://ch.promega.com/products/protein-detection/primary-and-secondary-antibodies/anti-halotag-pab/<br>anti-Myc (Cell Signaling, #2276S) https://www.cellsignal.co.uk/products/primary-antibodies/myc-tag-9b11-mouse-mab/2276<br>anti-Tubulin (Merck, #T6199) https://www.sigmaaldrich.com/US/en/product/SIGMA/T6199<br>anti-GAL9 (R&D Systems, #T6199) https://www.sigmaaldrich.com/US/en/product/SIGMA/T6199<br>anti-p62 (MBL, #PM045Y) https://www.mblintl.com/products/8485/<br>anti-LC3 (MBL, #PM036Y) https://www.mblintl.com/products/8485/<br>anti-RNF13 (Merck, #HPA064784) https://www.sigmaaldrich.com/US/en /product/SIGMA/HPA064784<br>anti-Ubiquitin (Santa Cruz, #sc-8017) https://www.scbt.com/p/ubiquitin-antibody-p4d1<br>anti-UBE1 (Santa Cruz, #sc-53555) https://www.scbt.com/p/ube1-antibody-2g2<br>anti-HBc (Kanto Chemical, #sc-53555) https://products.kanto.co.jp/web/index.cgi?c=t_product_table&pk=763

Eukaryotic cell lines

Policy information about cell lines

Cell line source(s)	HEK293 cells (ATCC), HepG2 cells (ATCC), HepG2 Tet-On Advanced cells (Takara Bio), Primary human hepatocytes (PhoenixBio).
Authentication	Cells obtained from ATCC, Takara Bio, and PhoenixBio were authenticated by the vendor using morphology and PCR based approaches.
Mycoplasma contamination	Cell lines used in this study were tested negative for mycoplasma (Lonza MycoAlert kit).
Commonly misidentified lines (See ICLAC register)	No commonly misidentified cell lines were utilized in this study.
